# Work‑Related Migration to the Alang Ship‑Breaking Industry from Other Parts of India: An Overview of Health‑Related Issues

**DOI:** 10.5334/aogh.4735

**Published:** 2025-06-12

**Authors:** Raja Singh, Arthur L Frank

**Affiliations:** 1 Post-Doctoral Fellow, Department of Environmental and Occupational Health, Dornsife School of Public Health, Drexel University, Philadelphia, PA, USA; 2 Clinical Professor and Professor Emeritus, Department of Environmental and Occupational Health, Dornsife School of Public Health, Drexel University, Philadelphia, PA, USA

**Keywords:** mesothelioma, asbestos, latency, Purvanchal, migration, public health, occupational health

## Abstract

*Background:* India has a ship‑breaking yard at Alang in Gujarat. The workers are prone to being exposed to various hazardous chemicals, including asbestos. These workers are often interstate migrants, and there is a chance of them developing diseases caused by asbestos, manifesting decades after exposure. This includes mesothelioma, which is a malignancy caused by asbestos exposure and can manifest much after the cessation of their employment.

*Objective:* In the absence of an operational national database of migrants, it is important to understand the source of migrants to trace future disease occurrence, especially after retiring to their home states. This study aims to find the Indian districts from which workers migrate to work at Alang‑Sosiya ship‑breaking yards.

*Methods:* The current study uses the Right to Information Act, 2005, to find out the districts of residents of migrant workers that came to Alang in 2019 as a representative year.

*Findings and conclusion:* The data point to districts in three states: Uttar Pradesh, Jharkhand and Bihar, and have important policy consequences and epidemiological importance as these can be used to understand the aetiology of asbestos‑related diseases.

## Background

Alang‑Sosiya in Bhavnagar district of the State of Gujarat has a ship‑breaking yard off the coast where end‑of‑life ships from across the world are dismantled for recycling. This area is often looked at due to the hazardous nature of the industry and because there is dismantling of old ships which would have hazardous materials, including asbestos, present. Asbestos can end up causing health issues and environmental damage. With respect to health and safety issues, there was a common colloquial term called ‘*Alang se Palang’* which roughly translates to ‘from Alang to the deathbed’, an indicator of the possibility of debilitating diseases occurring among workers who may be exposed to workplace hazards and hazardous chemicals, dismantling and recycling end‑of‑life ships. A total of 8,062 ships have been scrapped from 1982 to 2020 at Alang [1]. In the years 2006 to 2007, the National Institute of Occupational Health (NIOH) released a report which stated that there is *‘asbestos used in the hanger liners, mastic under insulation, cloth over insulation, cable, lagging and insulation on pipes and hull, adhesives, gaskets on piping connections, and valve packing, in a ship’* which are potential sources of exposure in ships during unsafe removal and recycling [2].

A study was performed by the NIOH where the records were supplied by the Directorate of Industrial Safety and Health, and medical examination records for asbestos handlers were identified by the Gujarat Maritime Board [2]. Out of 94 workers occupationally exposed to asbestos, 15 (16%) showed linear shadows on chest X‑rays and 26 workers (39%) showed restrictive impairment. In another study from a ship‑breaking yard in Sitakunda, Bangladesh, 94 workers were examined [3]. History of exposure and X‑rays were taken. Asbestosis was diagnosed in 33 workers (35%). Twenty‑five workers had a Forced Vital Capacity or FVC of less than 80% predicted and 23 (24%) had Forced Expiratory Volume FEV_1_ less than 80% predicted. However, neither study may have accounted for the decades‑long latency of malignancies like mesothelioma to manifest. Another study from Alang filled this gap and used the mesothelioma mortality risk as per a scientific projection model and found that 15% of the total workforce engaged will suffer from mesothelioma, which translates to 4,513 estimated cases [4]. However, looking at government records which another study investigated, there are zero reported cases of ‘occupational cancer’ (under which mesothelioma may be reported) under the Factories Act, 1948 from Gujarat [5]. In another study, which looked at cases over a four year period, 54 cases of mesothelioma (not distinguished as occupational or non‑occupational) were found in the National Cancer Registry Programme, while the study found 1,126 cases from just 83 public hospitals during the same period [6]. There is clearly an issue of poor cancer and occupational disease recordkeeping, which can also be explained in part by the possibility of misdiagnosis and/or under‑reporting. The Indian Supreme Court has mandated the medical records of staff working in the asbestos industry to be kept for 15 years after cessation of employment with the intention of keeping track of disease with long latency periods [7]. But this seems to be seldom followed (or may not even apply to cases of ship‑breaking yards by strict definition but may need to be considered) as there is a large proportion of migrant workers. There is not only migration in terms of people moving from one state to another to look for job opportunities but also migration in terms of people going to another state for medical care. From 10 year’s data, a substantial percentage (40.26%) of mesothelioma patients diagnosed at the Gujarat Cancer Research Institute, which is a premier cancer hospital in Ahmedabad, are from the adjoining states of Rajasthan and Madhya Pradesh [6]. At India’s premier cancer institute, i.e. the Tata Memorial Centre in Mumbai, the overall percentage of patients (total hospital registration) coming from Uttar Pradesh, Bihar and Jharkhand is 12.9%, 8.8% and 4.2%, respectively, with the total for the three states being 25.8% of the total registrations at the hospital [8]. This suggests that the health burden of the state where the workers are employed can sometimes be transferred to the state from which the workers came. Many cases will already be among those in states with lesser medical care facilities. Other states, where neither the workers came from nor where the industry where they worked, may be burdened because they simply may have a good medical facility. The current absence of an operational, fully populated database of the workers who work or go for treatment across states means that there may be an absence of important policy‑informing data available to policymakers. This may be with respect to the states where there may be a health burden post‑employment, especially due to long latency diseases. It is noted that in the home states of migrant workers, there may be no industry present. It is therefore important to understand the geographies from which people come to Alang for work, as there is a high possibility of asbestos exposure which will manifest into disease when this population returns to their home states. No known study was found which provides accurate information with respect to the areas in India from which workers come to work in the ship‑breaking industry in Alang, Gujarat, India. This is epidemiologically useful as it creates a link to further investigate the possible incidence of disease caused by asbestos exposure manifesting decades later, with a change in the location of workers by then. This can inform policy related to health, migration, cancer recordkeeping and labour welfare (including compensation).

## Aim

The aim of this paper is to find the Indian districts from which workers migrate to work at Alang‑Sosiya ship‑breaking yards.

## Methodology

Every worker, regardless of their employment status and type of job, including white‑collar workers, must undergo a 12 day ‘Comprehensive Safety Training’ programme. The number of people who undertook the training is a proxy for the number of workers who came into Alang‑Sosiya ship‑breaking yards for work, since such training is compulsory.

An information request under the Right to Information Act, 2005, was filed before the Gujarat Maritime Board, Gujarat (GMB) to obtain the number of people who were trained in 2019 for the ‘Comprehensive Safety Training’ [9]. The data were provided by GMB and collected. The year 2019 was before the COVID‑19 pandemic lockdowns and reflects more normal times. The district‑wise count of workers was collected and is presented in this study.

No names, personal details or identifiers were seen or recorded. Only the district and state were recorded, and a count made. The study does not involve any human participants, drugs, animals or human tissue, and includes non‑personal data available in the public domain and hence not under the purview of ethics clearance. The data used in the paper are in the public domain and are derived using the Right to Information Act, 2005, where personal information cannot be provided by law by public authorities (in this case, GMB), by default [10].

## Results

**Figure 1:**
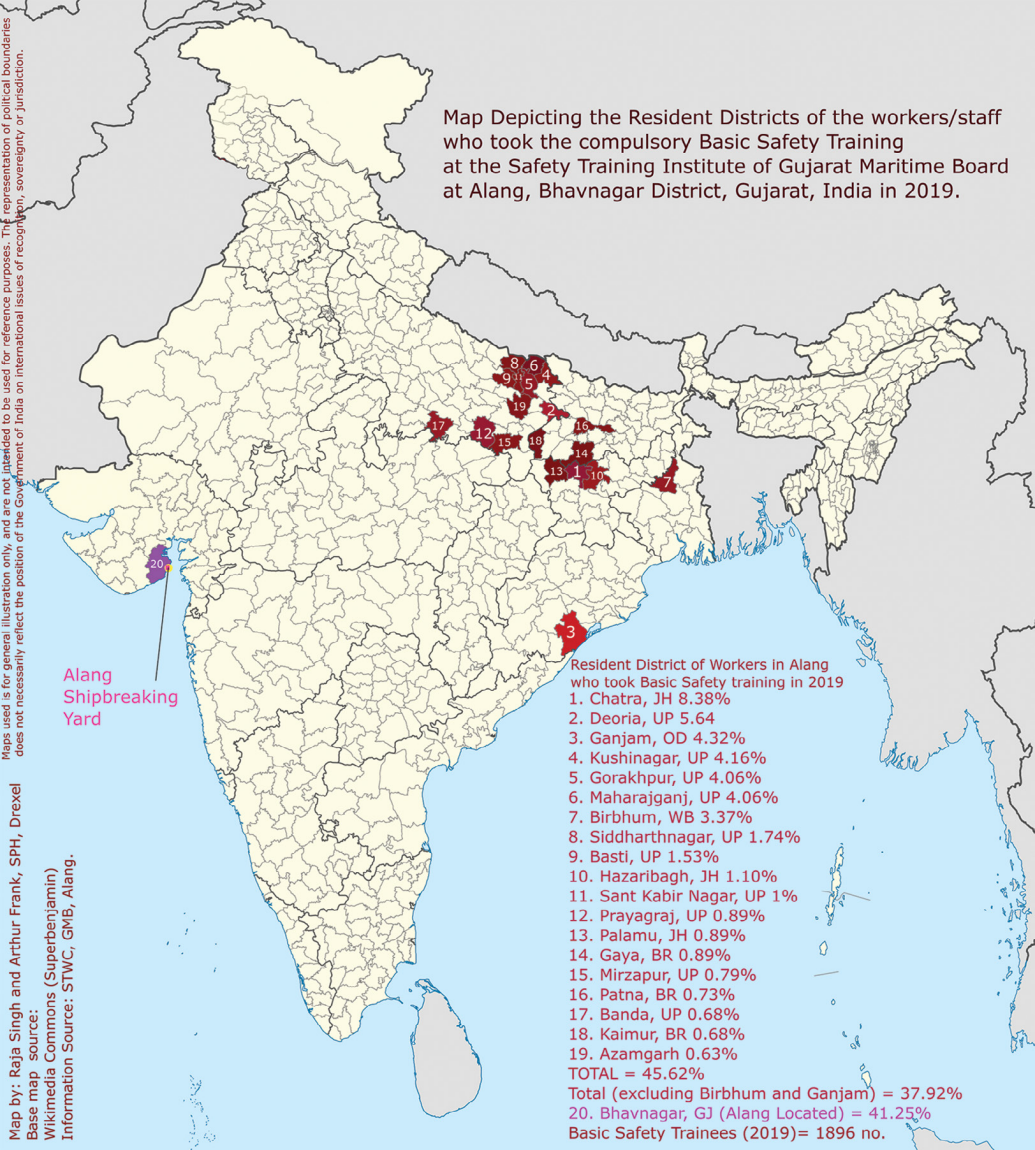
Map depicting the resident districts of the workers/staff who took the compulsory Basic ‘Comprehensive Safety Training’ at the Safety Institute of Gujarat Maritime Board at Alang, Bhavnagar District, Gujarat, India in 2019. Source: Author; attributed in picture.

Out of the 1,896 people who took the basic ‘Comprehensive Safety Training’ by GMB at the Alang training centre, the top districts for the number of people trained are given in Table 1 and represented graphically in Figure 1. A total of 37.9% of workers come from three states distant from Alang (Uttar Pradesh, Bihar and Jharkhand), which is a part of the larger cultural area of *Purvanchal* and allied areas [11].

**Table 1:** The percentage‑wise list of workers from the top districts who took the basic ‘Comprehensive Safety Training’ in 2019.

NAME OF THE DISTRICT	STATE IN INDIA	WORKER COUNT (%) (N = 1,896)
**Bhavnagar**	Gujarat	41.25
**Chatra**	Jharkhand	8.38
**Deoria**	Uttar Pradesh	5.64
**Ganjam**	Odisha	4.32
**Kushinagar**	Uttar Pradesh	4.16
**Gorakhpur**	Uttar Pradesh	4.06
**Maharajganj**	Uttar Pradesh	4.06
**Birbhum**	West Bengal	3.37
**Siddharthnagar**	Uttar Pradesh	1.74
**Basti**	Uttar Pradesh	1.53
**Hazaribagh**	Jharkhand	1.10
**Sant Kabir Nagar**	Uttar Pradesh	1.00
**Prayagraj**	Uttar Pradesh	0.89
**Palamu**	Jharkhand	0.89
**Gaya**	Bihar	0.89
**Mirzapur**	Uttar Pradesh	0.79
**Patna**	Bihar	0.73
**Banda**	Uttar Pradesh	0.68
**Kaimur**	Bihar	0.68
**Azamgarh**	Uttar Pradesh	0.63
**Others**		13.21

Further, other incidental data made available include the count of the total number of people trained in all the courses, apart from the basic ‘Comprehensive Safety Training’ programme, including ‘Gas Cutters Safety Training’ and ‘Refresher Comprehensive Safety Training for All’, which was a total of 5,972 people trained in 2019. The total number of people trained in all the courses from 2003 to 2023 (till October) is 156,709. This also means that prior to 2003, there was no formal training under the same mechanisms. The course includes seven modules which include: (a) Introduction to Ship Recycling, (b) Steel Metal Cutting, (c) Hazards in Ship Recycling & Material Handling, (d) Locational Hazards & Soft Skills, (e) Safety in Material Handling, (f) Fire & Explosion Safety and (g) First Aid & Emergency Awareness about Environment & Cleanliness.

## Discussion and Conclusion

This paper found the districts of various Indian states from which workers migrate to ship‑breaking yards in Alang‑Sosiya, Gujarat. This paper has used the methodology of using the Right to Information Act, 2005, to acquire information and has reported that apart from the district of Bhavnagar (where the Along‑Sosiya ship‑breaking yards are located), the majority of workers come from distinct pockets of the country, which includes a major part of eastern Uttar Pradesh, western Bihar, and northern parts of Jharkhand. A total of 37.9% of workers come from these three states, which form a common cultural area. A large part of this area is also referred to culturally as the *Purvanchal* area. The data are not surprising as these districts also have the largest migrant worker population in the country [12, p. 139]. Other areas where migrant workers come are districts from states of West Bengal and Odisha, but the larger number still comes from the *Purvanchal* Area as described above [11].

The larger issue is that there is interstate migration of labour in India, which means that many work as casual contract labour, which may not be fully covered under welfare‑oriented labour regulations. The Indian parliament in 1979 passed the Inter‑State Migrant Workmen (Regulation of Employment and Conditions of Service) Act, 1979. This welfare legislation regulates wage rates, holidays, hours of work and other conditions of service, and also provides for other allowances and provision of ‘prescribed medical facilities to the workmen, free of charge’. It also calls for the registration of the interstate migrant labour force. A study by the Tata Institute of Social Science in 2014 noted that ship‑breaking yards continue to recruit migrants without registering under the Inter‑State Migrant Workmen (Regulation of Employment and Conditions of Service) Act, 1979 [13]. With the best of intentions, the Government of India revamped labour laws and reduced the number of laws and consolidated them into four labour codes in 2020. For the welfare, occupational health and safety ‘the Occupational Safety, Health, and Working Conditions Code, 2020’ has been created. Provisions relating to interstate migrants were retained in this recently created code [14]. The important improvement was that it includes the creation of a database for interstate migrants. This new code also amalgamates the labour welfare measures of the Factories Act, 1948, which was the precursor to the Code. It is important that these well‑intentioned measures are fully implemented in letter and spirit.

Information from these three states is also important because two of these three states (Uttar Pradesh and Bihar) have not declared cancer as a notifiable disease, as is done in 17 other states in India. The Supreme Court of India has noted the long latency of asbestos‑related diseases, especially mesothelioma, and cases are expected in these two states [7, 15]. Mesothelioma, as a notifiable occupational disease, may be recorded as ‘Occupational Cancer’ under the Factories, Act 1948, but finds a separate classification under the notifiable diseases under the Mines Act, 1952 [16, 17]. The Supreme Court directed all industries dealing with asbestos to maintain medical records for 15 years after retirement or end of employment and this rule importantly should have validity in ship dismantling industries where the workers are directly or indirectly exposed to asbestos [2, 7]. It is relevant to study the epidemiological extent of mesothelioma in those states which have migration‑based economies and workers may return home after working elsewhere. This suggests further work in this area, especially in finding any relation between the current recordkeeping of mesothelioma and finding the aetiological link to industries where asbestos exposure may be present.

Further, even though health is a state subject, the issue of occupational disease recordkeeping, especially malignancies like mesothelioma, must be kept at a central level with a common patient identifier so that the workers’ medical records can be tracked even as they move from one state to another. Also appropriate is to have cancer become a notifiable disease nationwide and have all medical facilities linked to the national cancer registry program, and all cancers, occupational or not, be recorded in a common registry [18]. There is also a need for a dedicated occupational disease registry as the regulators under the Mines Act, 1952, and the Factories Act, 1948, seem to not be fully getting the actual incidence as the reporting may not be universal, and hospitals may be reporting cases through research publications, but not to the regulators [5, 19].

There are limitations to this study, which as persons trained under the basic ‘comprehensive safety training’, include white‑collar workers, this cannot be a full representation of all manual workers. There is another gas‑welder training programme, which may provide a more accurate estimation of the migrant labour, as it is a programme that does not include white‑collar workers. This could be the subject of an additional study. The data are only for a single year and do not fully represent other years in the past when workers may have been trained and continue to work. Similar data from other years would allow for a fuller picture of migrant workers’ issues.

India has also passed the Recycling of Ships Act, 2019, and this brought a stricter regime of environmental and health safety issues linked with the Hong Kong Convention [20]. The Hong Kong Convention, adopted in 2009, is aimed at ensuring ship‑breaking becomes safe for human health, safety and the environment [21]. This is an improvement over the Ship Breaking Code 2013 which was made in response to a Supreme Court judgement related to the environment, health and safety at Alang after a French ship Clemenceau, which had asbestos, was decided upon [22]. The new Act on recycling of ships is very conscious of the Basel Convention and restrictions on the movement of hazardous waste and also states that no ship shall install or use such prohibited hazardous materials, and there needs to be an inventory of hazardous materials aboard ships, before a ship is brought for recycling [20, 23]. The list of hazardous materials, as in the Recycling of Ship Rules, 2021, notified under Section 42 of the Recycling of Ships Act, 2019, has included asbestos and has set a threshold of 0.1% in materials, typically by weight [24]. This means that every shipowner needs to maintain an inventory of hazardous materials contained in the ship and keep this list updated throughout the life of the ship. This is in line with the Resolution of the Guidelines for the Development of the Inventory of Hazardous Materials [25]. Even though the International Labour Organization recommends the elimination of all future use of asbestos and asbestos‑containing materials, ships may have legacy asbestos waste that requires very stringent worker safety measures [26]. On the issue of the well‑intentioned labour welfare and environmental laws, what awaits is the full implementation of these well‑intentioned laws moving forward, and further studies are warranted on the implementation status of these regulations. Also needed are long‑term epidemiological studies which investigate the extent of mesothelioma and find its relation to certain exposures in workplaces. This is also in line with the recommendations of the parliamentary committee on cancer, which recommended studies on finding the causative factors of certain cancers and eliminating regional disparities in cancer prevalence [27].

## Authors Credit

Conceptualisation: RS; Data Curation: RS; Investigation: RS; Methodology: RS; Project Administration: ALF; Resources: ALF; Supervision: ALF; Validation: ALF; Writing, original draft: RS; Writing, review & editing: ALF, RS.

All authors had access to the data and a role in writing the manuscript.

## Declarations

Data is available as Supplementary material 1.

The work received no external funding.

The study does not involve any human participants, drugs, animals or human tissue, and includes non‑personal data available in the public domain and hence not under the purview of ethics clearance. The data used in the paper are in the public domain and are derived using the Right to Information Act, 2005, where personal information cannot be provided by law by public authorities, by default. The map of India, with its boundaries, in Figure 1 is used for general illustration only and is not intended to be used for reference purposes.

## Additional File

The additional file for this article can be found as follows:

Supplementary Material. The list of worker counts from the districts across India who took the basic ‘comprehensive safety training’ in 2019.
