# Supplementary material for: Work‑Related Migration to the Alang Ship‑Breaking Industry from Other Parts of India: An Overview of Health‑Related Issues
Source: Ann Glob Health. 2025 Jun 12;91(1):32. doi: 10.5334/aogh.4735 (PMC12164759; doi:10.5334/aogh.4735)
Supplement: Supplementary Material. — The list of worker counts from the districts across India who took the basic ‘comprehensive safety training’ in 2019. [file agh-91-1-4735-s1.pdf]

Title: Work-related migration to the Alang ship-breaking industry from other parts of India: An overview for health-related issues.
Authors: Singh, R and Frank, AL

Supplementary Material

Table: The list of worker count from the districts across India who took the basic 'comprehensive safety training' in 2019.

District Name, State Name	Count (n)	Count in % (n=1896)
**Bhavnagar, Gujarat**	**782**	**41.25**
**Chatra, Jharkhand**	159	8.39
**Deoria, Uttar Pradesh**	107	5.64
**Ganjam, Odisha**	82	4.32
**Kushinagar, Uttar Pradesh**	79	4.17
**Gorakhpur, Uttar Pradesh**	77	4.06
**Maharajganj, Uttar Pradesh**	77	4.06
**Birbhum, West Bengal**	64	3.38
**Siddharthnagar, Uttar Pradesh**	33	1.74
**Basti, Uttar Pradesh**	29	1.53
**Hazaribagh, Jharkhand**	21	1.11
**Sant Kabir Nagar, Uttar Pradesh**	19	1.00
**Prayagraj, Uttar Pradesh**	17	0.90
**Palamu, Jharkhand**	17	0.90
**Gaya, Bihar**	17	0.90
**Mirzapur, Uttar Pradesh**	15	0.79
**Patna, Bihar**	14	0.74
**Banda, Uttar Pradesh**	13	0.69
**Kaimur (Bhabua), Bihar**	13	0.69
**Azamgarh, Uttar Pradesh**	12	0.63
**Aurangabad, Uttar Pradesh**	9	0.47
**Munger, Bihar**	9	0.47
**Gonda, Uttar Pradesh**	8	0.42

**Rohtas, Bihar**	8	0.42
**Surat, Gujarat**	8	0.42
**Siwan, Bihar**	7	0.37
**Garhwa, Jharkhand**	6	0.32
**Jehanabad, Bihar**	6	0.32
**Anand, Gujarat**	6	0.32
**Mumbai, Maharashtra**	6	0.32
**Ambedkar Nagar, Uttar Pradesh**	5	0.26
**Kanpur Dehat, Uttar Pradesh**	5	0.26
**Shrawasti, Uttar Pradesh**	5	0.26
**Nalanda, Bihar**	5	0.26
**Kolkata, West Bengal**	5	0.26
**Bara Banki, Uttar Pradesh**	4	0.21
**Farrukhabad, Uttar Pradesh**	4	0.21
**Mau, Uttar Pradesh**	4	0.21
**Giridih, Jharkhand**	4	0.21
**Latehar, Jharkhand**	4	0.21
**Vaishali, Bihar**	4	0.21
**Ballia, Uttar Pradesh**	3	0.16
**Fatehpur, Uttar Pradesh**	3	0.16
**Chitrakoot, Madhya Pradesh**	3	0.16
**Jaunpur, Uttar Pradesh**	3	0.16
**Lakhimpur Kheri, Uttar Pradesh**	3	0.16
**Pratapgarh, Uttar Pradesh**	3	0.16
**Dhanbad, Jharkhand**	3	0.16
**Darbhanga, Bihar**	3	0.16
**Bardhaman (Both), West Bengal**	3	0.16
**Ludhiana, Punjab**	3	0.16

**Mallappuram, Kerala**	3	0.16
**Gurugram, Haryana**	3	0.16
**Bhiwani, Haryana**	3	0.16
**Badaun, Uttar Pradesh**	2	0.11
**Ghazipur, Uttar Pradesh**	2	0.11
**Ghaziabad, Uttar Pradesh**	2	0.11
**Unnao, Uttar Pradesh**	2	0.11
**Saran, Bihar**	2	0.11
**Amreli, Gujarat**	2	0.11
**Junagadh, Gujarat**	2	0.11
**Nagaur, Rajasthan**	2	0.11
**Satna, Madhya Pradesh**	2	0.11
**Dhar, Madhya Pradesh**	2	0.11
**Golaghata, West Bengal**	2	0.11
**Kannur, Kerala**	2	0.11
**Thiruvananthapuram, Kerala**	2	0.11
**Dehradun, Uttarkhand**	2	0.11
**Sangli, Maharashtra**	2	0.11
**Amethi, Uttar Pradesh**	1	0.05
**Bahraich, Uttar Pradesh**	1	0.05
**Bareilly, Uttar Pradesh**	1	0.05
**Bhadohi (Sant Ravidas Nagar), Uttar Pradesh**	1	0.05
**Chandauli, Uttar Pradesh**	1	0.05
**Etawah, Uttar Pradesh**	1	0.05
**Hamirpur, Uttar Pradesh**	1	0.05
**Hapur, Uttar Pradesh**	1	0.05
**Kannauj, Uttar Pradesh**	1	0.05
**Lucknow, Uttar Pradesh**	1	0.05

**Mainpuri, Uttar Pradesh**	1	0.05
**Meerut, Uttar Pradesh**	1	0.05
**Moradabad, Uttar Pradesh**	1	0.05
**Rampur, Uttar Pradesh**	1	0.05
**Shamli, Uttar Pradesh**	1	0.05
**Sitapur, Uttar Pradesh**	1	0.05
**Varanasi, Uttar Pradesh**	1	0.05
**Bokaro, Jharkhand**	1	0.05
**Ranchi, Jharkhand**	1	0.05
**Saraikela, Jharkhand**	1	0.05
**Lakhisarai, Bihar**	1	0.05
**Madhubani, Bihar**	1	0.05
**Muzzaffarpur, Bihar**	1	0.05
**Bhagalpur, Bihar**	1	0.05
**Bhojpur, Bihar**	1	0.05
**Champaran (Both), Bihar**	1	0.05
**Khagaria, Bihar**	1	0.05
**Sitamarhi, Bihar**	1	0.05
**Ahmedabad, Gujarat**	1	0.05
**Rajkot, Gujarat**	1	0.05
**Kutch, Gujarat**	1	0.05
**Devbhoomi Dwarka, Gujarat**	1	0.05
**Porbandar, Gujarat**	1	0.05
**Jamnagar, Gujarat**	1	0.05
**Jaipur, Rajasthan**	1	0.05
**Jhunjunu, Rajasthan**	1	0.05
**Sikar, Rajasthan**	1	0.05
**Sri Ganganagar, Rajasthan**	1	0.05

**Sawai Madhopur, Rajasthan**	1	0.05
**Naupada, Odisha**	1	0.05
**Khorda, Odisha**	1	0.05
**Sehore. Madhya Pradesh**	1	0.05
**Bhind. Madhya Pradesh**	1	0.05
**Indore, Madhya Pradesh**	1	0.05
**Koriya, Chhattisgarh**	1	0.05
**Sonitpur, Assam**	1	0.05
**Kamrup Metropolitan, Assam**	1	0.05
**Tarn Taran, Punjab**	1	0.05
**Pathankot, Punjab**	1	0.05
**Gurdaspur, Punjab**	1	0.05
**SBS Nagar, Punjab**	1	0.05
**Thrissur, Kerala**	1	0.05
**Ernakulam, Kerala**	1	0.05
**Kozhikode, Kerala**	1	0.05
**Pithorogarh, Uttarakhand**	1	0.05
**Hyderabad, Telangana**	1	0.05
**Tiruchirappalli, Tamil Nadu**	1	0.05
**Aurangabad, Maharashtra**	1	0.05
